# Association between physical activity levels and mild cognitive impairment in Chinese older adults: a cross-sectional study from the China health and retirement longitudinal study

**DOI:** 10.3389/fpubh.2025.1564544

**Published:** 2025-04-04

**Authors:** Lei Zhang, Wei Chen, Haijun Miao, Ting Zou, Xuan Xiang, Ruikai Wu, Xiaohui Zhou

**Affiliations:** Department of Geriatrics, First Affiliated Hospital of Xinjiang Medical University, Urumqi, China

**Keywords:** physical activity, mild cognitive impairment, older adults, China, CHARLS

## Abstract

**Background:**

Research indicates that mild cognitive impairment (MCI) in older adults is linked to physical activity; however, the relationship between varying levels of physical activity (PAL) and the risk of MCI needs further exploration.

**Objective:**

This study explores the association and dose–response relationship between different levels of physical activity and MCI in older adults.

**Methods:**

Using data from the 2020 China Health and Retirement Longitudinal Study (CHARLS), this cross-sectional analysis included 5,373 older adults aged 60 and above. Binary logistic regression models and restricted cubic spline (RCS) methods were employed to examine the association and dose–response relationship between different PAL levels and the risk of MCI in the overall population and subgroups. Sensitivity analyses were conducted to validate the robustness of the results.

**Results:**

In the overall study population, compared to the lowest PAL quartile, participants in the second PAL quartile had a significantly reduced risk of MCI by 21.3% (*p* < 0.05). Given that the second PAL quartile had the lowest risk of MCI, a logistic regression model was constructed using the second quartile as the reference group. The results showed that, compared to the second PAL quartile, participants in the first and fourth PAL quartiles had significantly increased risks of MCI by 27.1% (*p* < 0.05) and 38.2% (*p* < 0.05), respectively. In subgroup analyses, compared to the second PAL quartile, female participants in the third and fourth PAL quartiles had significantly increased risks of MCI by 50.1% (*p* < 0.05) and 89.0% (*p* < 0.05), respectively; participants aged 60–74 in the first and fourth PAL quartiles had significantly increased risks of MCI by 29.4% (*p* < 0.05) and 42.2% (*p* < 0.05), respectively; and rural residents in the fourth PAL quartile had a significantly increased risk of MCI by 33.5% (*p* < 0.05). In the Chinese older adult population, a dose–response relationship was observed between physical activity and the risk of MCI. The RCS curve showed that as physical activity increased, the risk of MCI gradually decreased, reaching a beneficial point at 900 MET-min/week, with the lowest risk at approximately 1,600 MET-min/week. Beyond 1,600 MET-min/week, the risk of MCI began to rise, reaching a significant increase at 2,100 MET-min/week. Sensitivity analyses confirmed the robustness of the findings.

**Conclusion:**

Physical activity levels between 900 and 2,100 MET-min/week are associated with a reduced risk of MCI in the Chinese older adult population. Using physical activity to predict the risk of MCI in this population is feasible, and moderate physical activity may be an effective strategy for preventing and managing MCI.

## 1 Introduction

With the intensification of global aging, cognitive dysfunction in older adults has become a major public health issue. Cognitive dysfunction not only leads to a decline in mental function and quality of life but also imposes a heavy disease and economic burden on patients and their families (1, 2). According to the World Alzheimer Report, 46.8 million people were living with dementia worldwide in 2015, and this number is projected to reach 131.5 million by 2050 (3). Alzheimer’s disease (AD) is the most common type of dementia (4), and primary prevention of AD holds significant potential, as one-third of global AD cases are attributed to modifiable risk factors. The WHO Guidelines for Reducing Cognitive Decline and Dementia Risk (5) and the 2020 Lancet Commission Report (6) emphasize that physical activity is a critical factor in dementia prevention. Mild cognitive impairment (MCI), which represents a transitional state between normal aging and dementia, is also a crucial “intervention window” for dementia prevention and treatment. Studies have shown (7) that up to 42.0% of older adults are affected by MCI. Data released by the Chinese National Health Commission indicate that there are approximately 15 million dementia patients aged 60 and above in China, of whom 10 million have AD, making China one of the fastest-growing countries in terms of AD prevalence (8). Jia et al. (8) found that there are 38.77 million MCI patients among individuals aged 60 and above in China, and it is estimated that the total population of MCI and dementia in adults aged 60 or older in China accounts for more than one-fifth of the global total.

Expert consensus and guidelines (9–18) indicate that physical activity is a Class I recommended measure for preventing and managing MCI. The American Academy of Neurology recommends exercise interventions as an effective method to enhance cognitive function in MCI patients. The 2023 international guidelines further emphasize that managing MCI through physical activity and exercise is one of the current key strategies. As a modifiable lifestyle factor, physical activity can reduce age-related cognitive decline (19–21). Two critical brain regions, the prefrontal cortex and the hippocampus, govern learning and memory functions. Research shows that with aging, the annual atrophy rate of white matter in the hippocampus and prefrontal cortex increases by 1–2%, and the reduction in their volume raises the risk of MCI. Studies have demonstrated that 1–2 years of regular, moderate physical exercise can increase hippocampal volume by 2%, and even 6 months after cessation of exercise, the sustained effects on cognitive function maintenance persist. Physical activity also promotes brain remodeling through multiple pathways, regulates neuroinflammation, facilitates neural circuit reorganization, and improves overall cognitive function in older adults. The primary mechanisms by which physical activity reduces the risk of cognitive impairment include increased cerebral blood flow, promotion of neurogenesis, synaptic plasticity, reduction of β-amyloid deposition, inhibition of neuroinflammation, and mitigation of oxidative stress-induced cellular damage (22). Kim et al. found (23) that sustained physical activity in MCI patients is associated with a reduced risk of Alzheimer’s-type dementia (more than 5 days of moderate-intensity physical activity per week or more than 3 days of vigorous-intensity physical activity per week).

The WHO recommends that older adults with normal cognitive function and those with mild cognitive impairment engage in physical exercise to reduce the risk of cognitive decline. It is recommended that adults aged 65 and above engage in at least 150 min of moderate-intensity aerobic activity, or 75 min of vigorous-intensity aerobic activity, or an equivalent combination of both, to achieve a weekly physical activity level of 600 MET-minutes (24, 25). Previous studies have suggested that different levels of physical activity benefit cognitive function in older adults (18, 26–32). However, recent findings from The Lancet (33) indicate that while physical activity benefits cognitive function in older adults, higher intensity and longer duration of physical activity do not necessarily lead to greater cognitive benefits, and high-intensity physical labor may even increase the risk of dementia. Additionally, the relationship between physical activity (PAL) and cognitive function may be influenced by gender and residential location, with PAL having a greater impact on cognitive function in older women than in older men. Given the lack of consensus in previous studies regarding the frequency, duration, and weekly energy expenditure of physical activity, this study investigates the relationship between PAL and MCI in Chinese older adults, aiming to establish recommended PAL levels and provide a scientific basis for the prevention and management of MCI in this population.

## 2 Methods

### 2.1 Data source and participants

**Figure 1:**
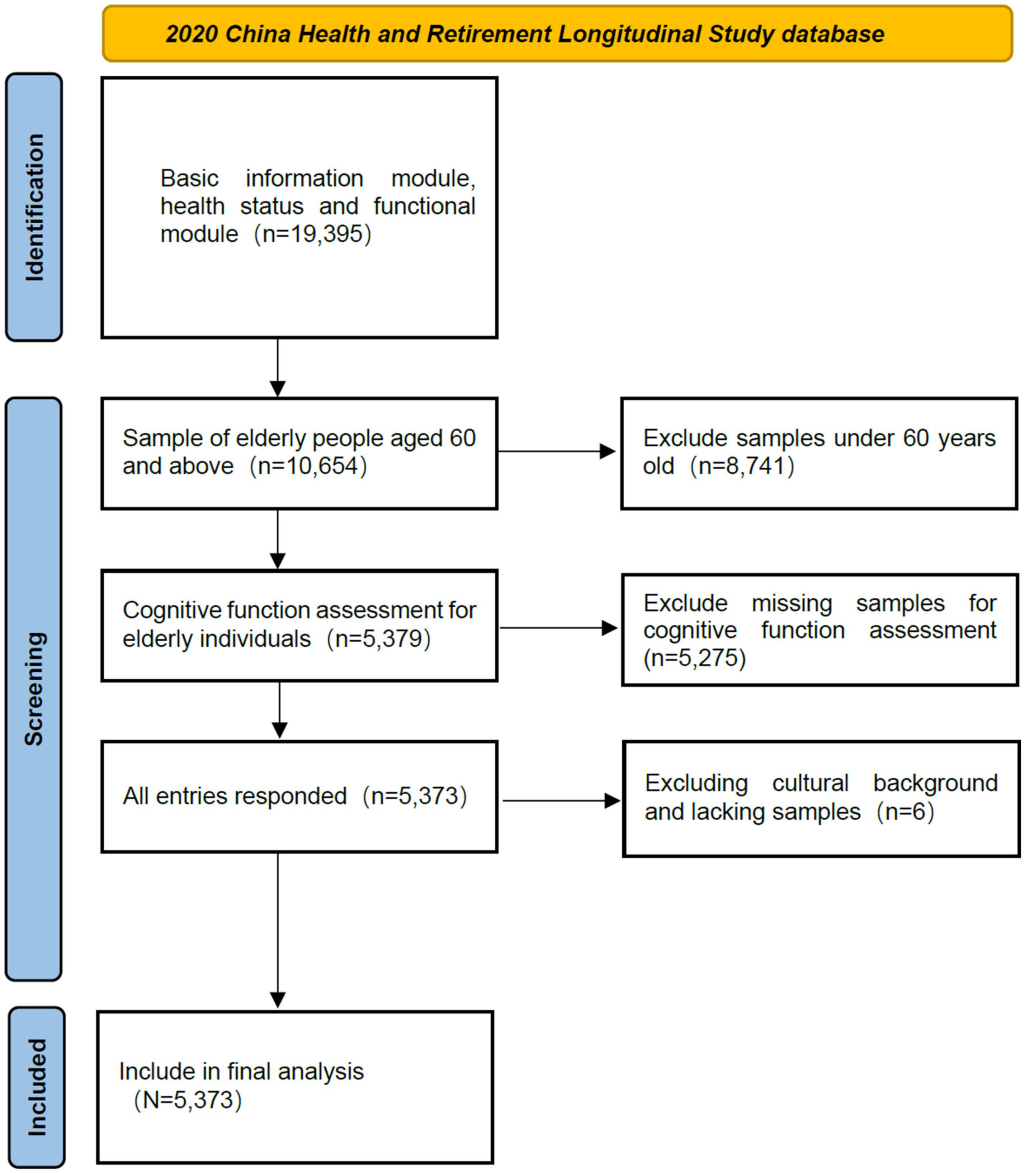
Flowchart of inclusion and exclusion.

The data for this study were obtained from the fifth wave of the China Health and Retirement Longitudinal Study (CHARLS), which was publicly released on November 16, 2023.^1^ CHARLS is a nationally representative survey of middle-aged and older adults in China, using a stratified, multi-stage, probability sampling method proportional to population size. The CHARLS questionnaire includes various modules, such as socio-demographic data, health and functional status, providing reliable data for exploring the relationship between physical activity and MCI in Chinese older adults. Additionally, the Biomedical Ethics Review Committee of Peking University (approval number: IRB00001052-11015) granted ethical approval for CHARLS (34). After obtaining authorization, the data were downloaded for this study. According to the definition of older adults in China, respondents aged 60 and above were included in the study. Eligible participants had complete data on physical activity, MCI status, and relevant covariates. Therefore, respondents under 60 or with missing data in these areas were excluded. A total of 5,373 participants were included in this study (see Figure 1).

### 2.2 Mild cognitive impairment

The cognitive function assessment in CHARLS follows the method used in the U.S. Health and Retirement Study (HRS). Participants were assessed face-to-face in four dimensions of cognitive function: orientation, memory, calculation, and drawing. For orientation and calculation, a telephone interview for cognitive status was used. Orientation was assessed by asking participants about the year, month, day, day of the week, and current season, with each correct answer scoring 1 point, for a total of 5 points. Calculation ability was assessed by asking participants to subtract 7 from 100 sequentially, repeating this process five times, with each correct calculation scoring 1 point, for a total of 5 points. Memory assessment included immediate recall of 10 randomly presented words, with each correct recall scoring 1 point. Additionally, delayed recall was assessed after participants completed the survey, calculation, and drawing tests. The total memory score was the sum of the scores from immediate and delayed word recall, with each correctly recalled word scoring 1 point. The total cognitive score was calculated as the sum of the scores from all four dimensions: orientation (5 points), calculation (5 points), memory (20 points), and drawing (1 point), with a maximum score of 31 points (35). This study defined MCI based on age-associated cognitive decline (AACD) (36). Specifically, older adults whose cognitive performance was at least 1 standard deviation (SD) below the age-specific standard were classified as having MCI. Participants aged 60 and above were divided into six-year age groups, and within each age group, those meeting the AACD criteria (i.e., scoring at least 1 SD below the age-specific standard) were identified as having MCI (37–41).

### 2.3 Physical activity assessment

The CHARLS database collected information on the activities respondents typically engaged in each week, including the number of days they performed activities lasting at least 10 min and the duration of daily activities (questionnaire codes: DA032–036). Physical activity levels were categorized as: inactive, light physical activity (e.g., walking, including walking at work or home, and walking for leisure, sports, exercise, or recreation), moderate-intensity physical activity (activities that make you breathe somewhat harder than normal, such as carrying light loads, cycling at a regular pace, mopping floors, practicing Tai Chi, or brisk walking), and vigorous-intensity physical activity (activities that make you breathe much harder than normal, such as carrying heavy loads, digging, farming, aerobic exercise, fast cycling, or cycling with a load). The CHARLS database did not record the exact duration of physical activity but instead used intervals. This study used the midpoint of each interval as the daily physical activity duration. The International Physical Activity Questionnaire-Short Form (IPAQ-SF) was used to assess physical activity (42). Metabolic equivalent (MET) values were assigned to each physical activity using the IPAQ, a widely used tool for measuring adult PAL, which has demonstrated reliability and validity (42). MET values of 3.3, 4.0, and 8.0 were assigned to light, moderate, and vigorous activities, respectively. Total weekly energy expenditure was calculated using the following formula: MET × daily activity duration (minutes) × weekly activity days (days) (43). Physical activity levels were divided into quartiles for analysis: first quartile (<720 MET-min/week), second quartile (720 ≤ ~ < 2,100 MET-min/week), third quartile (2,100 ≤ ~ < 5,040 MET-min/week), and fourth quartile (≥5,040 MET-min/week).

### 2.4 Covariates

Covariates in this study were selected based on their potential impact on MCI. Socio-demographic data, health status, and other indices were extracted from the 2020 CHARLS database as independent variables. Variables were assessed according to their type, and values were assigned accordingly for categorical variables. For binary categorical variables, one category was assigned a value of 0, and the other category was assigned a value of 1. For categorical variables with three or more categories, incremental values (e.g., 0, 1, 2, etc.) were assigned to represent each category. The specific variables selected for assessment were as follows:

1. Demographic characteristics: age, residential location, gender, education level, marital status.
2. Health behaviors: smoking, alcohol consumption.
3. Chronic diseases: hypertension, dyslipidemia (high or low blood lipids), diabetes or elevated blood sugar (including impaired glucose tolerance and elevated fasting blood sugar), malignant tumors (excluding mild skin cancer), chronic lung diseases [e.g., chronic bronchitis or emphysema, pulmonary heart disease (excluding tumors or cancer)], liver diseases (excluding fatty liver, tumors, or cancer), heart diseases (e.g., myocardial infarction, coronary heart disease, angina pectoris, congestive heart failure, and other heart diseases), stroke, kidney diseases (excluding tumors or cancer), stomach or digestive system diseases (excluding tumors or cancer), emotional and mental issues, memory-related diseases (e.g., dementia, brain atrophy), Parkinson’s disease, arthritis or rheumatism, asthma (non-pulmonary disease), depression.

### 2.5 Data analysis

Statistical analyses were performed using R version 4.3.0. Continuous variables that did not follow a normal distribution were expressed as medians (interquartile ranges) [M (*P_25_*, *P_75_*)], and group comparisons were conducted using non-parametric tests. Categorical data were expressed as *n* (%), and group comparisons were conducted using chi-square tests or Fisher’s exact tests. Binary logistic regression analysis was used to examine the relationship between different PAL levels and MCI in older adults. Model 1 was unadjusted for any variables; Model 2 was adjusted for socio-demographic characteristics and health behaviors that showed significant differences; Model 3 was adjusted for socio-demographic characteristics, health behaviors, and chronic diseases that showed significant differences. Subgroup analyses by gender, age, and residential location were conducted to explore differences between subgroups. Restricted cubic spline (RCS) analysis was used to assess the dose–response relationship between PAL and MCI in older adults. Results were expressed as odds ratios (ORs) with 95% confidence intervals (CIs), and differences were considered statistically significant when *p* < 0.05.

## 3 Results

### 3.1 Basic characteristics of participants

Table 1 shows the baseline characteristics of the 5,373 older adult participants. The mean age was 67.49 ± 5.55 years, with 3,161 males (58.83%) and 2,212 females (41.17%). Among the participants, 847 (15.76%) were identified as having MCI based on cognitive assessment. Significant differences (*p* < 0.05) were observed between the non-MCI and MCI groups in weekly total energy expenditure, age, residential location, gender, marital status, education level, alcohol consumption, stroke, memory-related diseases (e.g., dementia, brain atrophy), and depression. No significant differences (*p* > 0.05) were found in smoking, dyslipidemia (high or low blood lipids), diabetes or elevated blood sugar (including impaired glucose tolerance and elevated fasting blood sugar), malignant tumors (excluding mild skin cancer), chronic lung diseases [e.g., chronic bronchitis or emphysema, pulmonary heart disease (excluding tumors or cancer)], liver diseases (excluding fatty liver, tumors, or cancer), heart diseases (e.g., myocardial infarction, coronary heart disease, angina pectoris, congestive heart failure, and other heart diseases), kidney diseases (excluding tumors or cancer), stomach or digestive system diseases (excluding tumors or cancer), emotional and mental issues, Parkinson’s disease, arthritis or rheumatism, and asthma (non-pulmonary disease).

**Table 1:** Basic characteristics of participants.

Variables	All (*n* = 5,373)	Non-MCI group (*n* = 4,526)	MCI group (*n* = 847)	Statistic	*P*
Physical activity level, *n*(%)				*χ*^2^ = 31.70	**<0.001**
Q1	1,335(24.85)	1,114(24.61)	221(26.09)		
Q2	1,337(24.88)	1,173(25.92)	164(19.36)		
Q3	1,320(24.57)	1,131(24.99)	189(22.32)		
Q4	1,381(25.70)	1,108(24.48)	273(32.23)		
Age, *M* (*P_25_*, *P_75_*)	66.00(63.00,71.00)	66.00(63.00,71.00)	67.00(63.00,71.00)	*Z* = −2.30	**0.022**
Residential location, *n*(%)				*χ*^2^ = 91.54	**<0.001**
Countryside	3,761(70.00)	3,051(67.41)	710(83.83)		
City	1,612(30.00)	1,475(32.59)	137(16.17)		
Gender, *n*(%)				*χ*^2^ = 7.21	**0.007**
Male	3,161(58.83)	2,698(59.61)	463(54.66)		
Female	2,212(41.17)	1,828(40.39)	384(45.34)		
Marriage, *n*(%)				*χ*^2^ = 19.20	**<0.001**
Married, living with spouse	4,235(78.82)	3,599(79.52)	636(75.09)		
Married, but temporarily not living with spouse due to work or other reasons	242(4.50)	206(4.55)	36(4.25)		
Separation (no longer living together as spouses)	21(0.39)	13(0.29)	8(0.94)		
Divorce	57(1.06)	51(1.13)	6(0.71)		
Widowed or widowed	797(14.83)	641(14.16)	156(18.42)		
Never married	21(0.39)	16(0.35)	5(0.59)		
Level of education, *n*(%)				*χ*^2^ = 303.22	**<0.001**
Illiterate	624 (11.61)	413 (9.13)	211 (24.91)		
Elementary school	2673 (49.75)	2173 (48.01)	500 (59.03)		
Junior high school and above	2076 (38.64)	1940 (42.86)	136 (16.06)		
Smoking, *n*(%)				*χ*^2^ = 1.62	0.203
No	3,809(70.89)	3,224(71.23)	585(69.07)		
Yes	1,564(29.11)	1,302(28.77)	262(30.93)		
Drinking alcohol, *n*(%)				*χ*^2^ = 15.02	**<0.001**
No	3,270(60.86)	2,704(59.74)	566(66.82)		
Yes	2,103(39.14)	1,822(40.26)	281(33.18)		
Hypertension, *n*(%)				*χ*^2^ = 1.45	0.228
No	4,982(92.72)	4,205(92.91)	777(91.74)		
Yes	391(7.28)	321(7.09)	70(8.26)		
Abnormal blood lipids (high or low blood lipids), *n*(%)				*χ*^2^ = 3.27	0.071
No	4,919(91.55)	4,157(91.85)	762(89.96)		
Yes	454(8.45)	369(8.15)	85(10.04)		
Diabetes or elevated blood sugar (including abnormal glucose tolerance and elevated fasting blood sugar), *n*(%)				*χ*^2^ = 0.34	0.56
No	5,115(95.20)	4,312(95.27)	803(94.81)		
Yes	258(4.80)	214(4.73)	44(5.19)		
Malignant tumors such as cancer (excluding mild skin cancer), *n*(%)				*χ*^2^ = 0.00	0.949
No	5,317(98.96)	4,479(98.96)	838(98.94)		
Yes	56(1.04)	47(1.04)	9(1.06)		
Chronic lung diseases such as chronic bronchitis or emphysema, pulmonary heart disease (excluding tumors or cancer), *n*(%)				*χ*^2^ = 0.42	0.519
No	5,105(95.01)	4,304(95.10)	801(94.57)		
Yes	268(4.99)	222(4.90)	46(5.43)		
Liver diseases (excluding fatty liver, tumors, or cancer), *n*(%)				*χ*^2^ = 0.01	0.907
No	5,224(97.23)	4,401(97.24)	823(97.17)		
Yes	149(2.77)	125(2.76)	24(2.83)		
Heart disease (such as myocardial infarction, coronary heart disease, angina pectoris, congestive heart failure, and other heart diseases), *n*(%)				*χ*^2^ = 1.56	0.211
No	5,043(93.86)	4,240(93.68)	803(94.81)		
Yes	330(6.14)	286(6.32)	44(5.19)		
Stroke, *n*(%)				*χ*^2^ = 6.05	**0.014**
No	5,246(97.64)	4,429(97.86)	817(96.46)		
Yes	127(2.36)	97(2.14)	30(3.54)		
Kidney disease (excluding tumors or cancer), *n*(%)				*χ*^2^ = 0.79	0.373
No	5,146(95.78)	4,330(95.67)	816(96.34)		
Yes	227(4.22)	196(4.33)	31(3.66)		
Diseases of the stomach or digestive system (excluding tumors or cancer), *n*(%)				*χ*^2^ = 1.29	0.256
No	5,082(94.58)	4,274(94.43)	808(95.40)		
Yes	291(5.42)	252(5.57)	39(4.60)		
Emotional and mental issues, *n*(%)				*χ*^2^ = 2.32	0.128
No	5,327(99.14)	4,491(99.23)	836(98.70)		
Yes	46(0.86)	35(0.77)	11(1.30)		
Diseases related to memory (senile dementia, brain atrophy), *n*(%)				*χ*^2^ = 8.24	**0.004**
No	5,110(95.11)	4,321(95.47)	789(93.15)		
Yes	263(4.89)	205(4.53)	58(6.85)		
Parkinson’s disease, *n*(%)				*χ*^2^ = 0.33	0.568
No	5,325(99.11)	4,487(99.14)	838(98.94)		
Yes	48(0.89)	39(0.86)	9(1.06)		
Arthritis or rheumatism, *n*(%)				*χ*^2^ = 0.02	0.893
No	5,044(93.88)	4,248(93.86)	796(93.98)		
Yes	329(6.12)	278(6.14)	51(6.02)		
Asthma (non-pulmonary disease), *n*(%)				*χ*^2^ = 0.00	0.994
No	5,259(97.88)	4,430(97.88)	829(97.87)		
Yes	114(2.12)	96(2.12)	18(2.13)		
Depression, *n*(%)				*χ*^2^ = 153.77	**<0.001**
No	3,358(62.50)	2,989(66.04)	369(43.57)		
Yes	2,015(37.50)	1,537(33.96)	478(56.43)		

Q, Quartile. Bold value: *p* < 0.05, the difference is statistically significant.

### 3.2 Binary logistic regression analysis of physical activity and MCI

This study used MCI as the dependent variable and physical activity as the independent variable, with other covariates included in the regression models to assess their relationships. The variance inflation factor (VIF) for multicollinearity testing was <5, and tolerance was >0.1, indicating no multicollinearity among the included independent variables. Multivariate logistic regression analysis was performed, and the results are shown in Table 2. The first logistic regression model included only PAL, showing that compared to the lowest PAL quartile, participants in the second PAL quartile had a 29.5% reduced risk of MCI [*OR* = 0.705, 95%*CI* (0.567, 0.876), *p* < 0.05], while participants in the fourth PAL quartile had a 24.2% increased risk of MCI [*OR* = 1.242, 95%*CI* (1.021, 1.511), *p* < 0.05].

**Table 2:** Binary logistic regression analysis of the association between physical activity and MCI.

Variable	Subgroup	Model 1	Model 2	Model 3
PAL				
	Q1	1.000(Reference)	1.000(Reference)	1.000(Reference)
	Q2	0.705*(0.567 ~ 0.876)	0.789*(0.629 ~ 0.989)	0.787*(0.626 ~ 0.989)
	Q3	0.842(0.682 ~ 1.040)	0.877(0.705 ~ 1.092)	0.895(0.717 ~ 1.116)
	Q4	1.242*(1.021 ~ 1.511)	1.085(0.882 ~ 1.333)	1.087(0.882 ~ 1.339)
PAL				
	Q2	1.000(Reference)	1.000(Reference)	1.000(Reference)
	Q1	1.419*(1.141 ~ 1.765)	1.267*(1.011 ~ 1.589)	1.271*(1.011 ~ 1.597)
	Q3	1.195(0.955 ~ 1.496)	1.112(0.881 ~ 1.403)	1.137(0.899 ~ 1.439)
	Q4	1.762*(1.428 ~ 2.175)	1.375*(1.102 ~ 1.716)	1.382*(1.104 ~ 1.729)

Data are presented as odds ratios (ORs) with 95% confidence intervals (CIs). Q, quartile. *p* < 0.05. Model 1: Unadjusted for any variables. Model 2: Adjusted for socio-demographic characteristics (age, residential location, gender, marital status, education level) and health behaviors (alcohol consumption) that showed significant differences. Model 3: Adjusted for socio-demographic characteristics (age, residential location, gender, marital status, education level), health behaviors (alcohol consumption), and chronic diseases [stroke, memory-related diseases (e.g., dementia, brain atrophy), depression] that showed significant differences.

The second logistic regression model adjusted for socio-demographic characteristics (age, residential location, gender, marital status, education level) and health behaviors (alcohol consumption) that showed significant differences. The results showed that compared to the lowest PAL quartile, participants in the second PAL quartile had a 21.1% reduced risk of MCI [*OR* = 0.789, 95%*CI* (0.629, 0.989), *p* < 0.05].

The third logistic regression model adjusted for socio-demographic characteristics (age, residential location, gender, marital status, education level), health behaviors (alcohol consumption), and chronic diseases [stroke, memory-related diseases (e.g., dementia, brain atrophy), depression] that showed significant differences. The results showed that compared to the lowest PAL quartile, participants in the second PAL quartile had a 21.3% reduced risk of MCI [*OR* = 0.787, 95%*CI* (0.626, 0.989), *p* < 0.05].

Given that the second PAL quartile had the lowest risk of MCI, a logistic regression model was constructed using the second quartile as the reference group. The third logistic regression model showed that compared to the second PAL quartile, participants in the first and fourth PAL quartiles had a 27.1% [*OR* = 1.271, 95%*CI* (1.011, 1.597), *p* < 0.05] and 38.2% [*OR* = 1.382, 95%*CI* (1.104, 1.729), *p* < 0.05] increased risk of MCI, respectively.

In binary logistic regression analysis, confounding variables may affect the association between PAL and MCI, potentially biasing the results. Therefore, this study assessed the robustness of the results by sequentially excluding these confounding factors and constructing three logistic regression models. Sensitivity analysis confirmed that the relationship between PAL and MCI remained robust.

**Figure 2:**
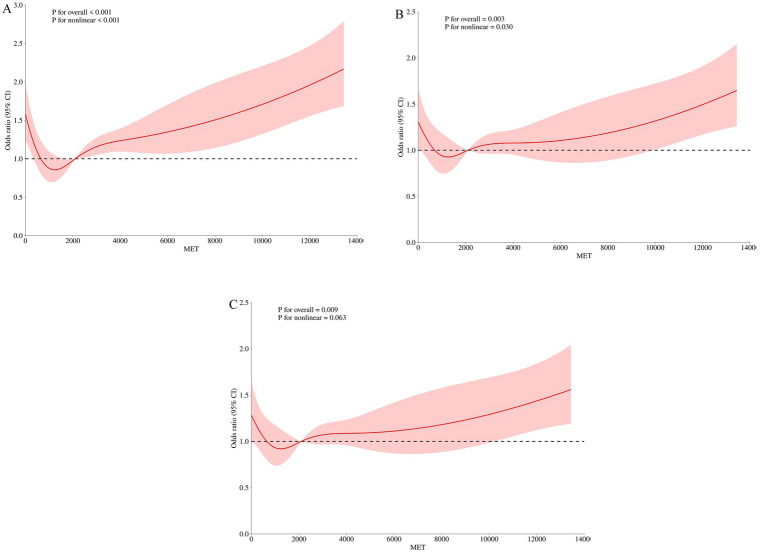
Dose–response relationship between physical activity and MCI in older adults. **(A)** Unadjusted for any variables. **(B)** Adjusted for socio-demographic characteristics (age, residential location, gender, marital status, education level) and health behaviors (alcohol consumption) that showed significant differences. **(C)** Adjusted for socio-demographic characteristics (age, residential location, gender, marital status, education level), health behaviors (alcohol consumption), and chronic diseases [stroke, memory-related diseases (e.g., dementia, brain atrophy), depression] that showed significant differences.

In this study, physical activity was introduced as a continuous variable in the restricted cubic spline (RCS) curve fitting. The RCS curve showed a linear relationship between physical activity and the risk of MCI in older adults (*P_overall_* = 0.009, *P_nonlinear_* = 0.063) (see Figure 2C). As physical activity increased, the risk of MCI gradually decreased, reaching a beneficial point at 900 MET-min/week, with the lowest risk at approximately 1,600 MET-min/week. Beyond 1,600 MET-min/week, the risk of MCI began to rise, reaching a significant increase at 2,100 MET-min/week. In RCS analysis, confounding variables may affect the dose–response relationship between physical activity and the risk of MCI. The study assessed the robustness of the results by sequentially including these confounding factors, and sensitivity analysis confirmed that the relationship between physical activity and MCI remained robust (see Figures 2A, B).

### 3.3 Subgroup analysis

This study conducted subgroup analyses to examine the effects of gender, age, and residential location on the risk of MCI in older adults. Age was divided into two groups: 60–74 and 75+, to investigate the relationship between physical activity and the incidence of diabetes in different age ranges.

Further subgroup analyses were conducted to explore the association between physical activity and MCI in older adults (Table 3). The first logistic regression model showed that compared to the second PAL quartile, male participants in the first and fourth PAL quartiles had a 37.3% (*p* < 0.05) and 43.2% (*p* < 0.05) increased risk of MCI, respectively. The third logistic regression model showed that compared to the second PAL quartile, female participants in the third and fourth PAL quartiles had a 50.1% (*p* < 0.05) and 89.0% (*p* < 0.05) increased risk of MCI, respectively. Participants aged 60–74 in the first and fourth PAL quartiles had a 29.4% (*p* < 0.05) and 42.2% (*p* < 0.05) increased risk of MCI, respectively. Rural residents in the fourth PAL quartile had a 33.5% (*p* < 0.05) increased risk of MCI.

**Table 3:** Logistic regression analysis of the relationship between PAL and MCI in subgroup older adults.

Variable	Pal	Model 1	Model 2	Model 3
Gender				
Male				
	Q2	1.000(Reference)	1.000(Reference)	1.000(Reference)
	Q1	1.373*(1.033 ~ 1.824)	1.292(0.962 ~ 1.735)	1.276(0.948 ~ 1.719)
	Q3	0.947(0.699 ~ 1.284)	0.895(0.653 ~ 1.226)	0.911(0.663 ~ 1.252)
	Q4	1.432*(1.091 ~ 1.879)	1.152(0.865 ~ 1.533)	1.127(0.844 ~ 1.506)
Female				
	Q2	1.000(Reference)	1.000(Reference)	1.000(Reference)
	Q1	1.494*(1.062 ~ 2.103)	1.295(0.909 ~ 1.846)	1.320(0.921 ~ 1.892)
	Q3	1.559*(1.109 ~ 2.192)	1.453*(1.021 ~ 2.068)	1.501*(1.048 ~ 2.149)
	Q4	2.432*(1.741 ~ 3.398)	1.792*(1.260 ~ 2.550)	1.890*(1.321 ~ 2.704)
Age				
60–74 years old				
	Q2	1.000(Reference)	1.000(Reference)	1.000(Reference)
	Q1	1.440*(1.131 ~ 1.832)	1.306*(1.017 ~ 1.677)	1.294*(1.006 ~ 1.665)
	Q3	1.214(0.949 ~ 1.553)	1.131(0.876 ~ 1.459)	1.140(0.881 ~ 1.474)
	Q4	1.873*(1.492 ~ 2.352)	1.437*(1.132 ~ 1.823)	1.422*(1.118 ~ 1.808)
≥75 years old				
	Q2	1.000(Reference)	1.000(Reference)	1.000(Reference)
	Q1	1.344(0.805 ~ 2.243)	1.222(0.711 ~ 2.101)	1.404(0.799 ~ 2.468)
	Q3	1.177(0.677 ~ 2.048)	1.070(0.600 ~ 1.908)	1.295(0.707 ~ 2.372)
	Q4	1.171(0.616 ~ 2.228)	0.949(0.483 ~ 1.867)	1.182(0.588 ~ 2.379)
Residence				
Rural area				
	Q2	1.000(Reference)	1.000(Reference)	1.000(Reference)
	Q1	1.313*(1.025 ~ 1.682)	1.244(0.964 ~ 1.606)	1.239(0.958 ~ 1.602)
	Q3	1.113(0.860 ~ 1.440)	1.067(0.818 ~ 1.390)	1.077(0.824 ~ 1.407)
	Q4	1.458*(1.154 ~ 1.842)	1.342*(1.053 ~ 1.711)	1.335*(1.045 ~ 1.705)
City				
	Q2	1.000(Reference)	1.000(Reference)	1.000(Reference)
	Q1	1.467(0.909 ~ 2.370)	1.377(0.841 ~ 2.255)	1.380(0.831 ~ 2.292)
	Q3	1.371(0.854 ~ 2.201)	1.300(0.799 ~ 2.117)	1.437(0.869 ~ 2.374)
	Q4	1.669(0.958 ~ 2.906)	1.500(0.840 ~ 2.677)	1.575(0.868 ~ 2.860)

Data are presented as odds ratios (ORs) with 95% confidence intervals (CIs). Q, quartile. *P* < 0.05. Model 1: Unadjusted for any variables. Model 2: Adjusted for socio-demographic characteristics (age, residential location, gender, marital status, education level) and health behaviors (alcohol consumption) that showed significant differences. Model 3: Adjusted for socio-demographic characteristics (age, residential location, gender, marital status, education level), health behaviors (alcohol consumption), and chronic diseases [stroke, memory-related diseases (e.g., dementia, brain atrophy), depression] that showed significant differences. Subgroup analyses in Models 2 and 3 did not include the main variable.

## 4 Discussion

With the further development of global aging and increasing life expectancy, dementia imposes a heavy disease and economic burden on older adults. AD is the most common type of dementia and a major global public health issue (44). The treatment costs for AD and related dementia are high (45, 46). U.S. statistics show that dementia patients spent over $387,000 per person in the past 5 years (47), and it is predicted that the number of dementia patients will double in the coming decades, with the time, financial, human, and material resources required for dementia care expected to increase. Although there is no cure for dementia, studies have found that 3% of dementia cases can be prevented by increasing physical activity in daily life (48, 49). Increasing evidence suggests that regular physical activity is associated with a reduced rate of dementia conversion in MCI patients, with a 15% reduction in dementia conversion rate among MCI patients who engage in regular physical activity (23, 50). Physical activity can prevent MCI and reduce the conversion from MCI to dementia, and it is the lowest-cost intervention. Therefore, early detection and intervention in MCI progression can help reduce the associated burden, benefiting individuals, families, and society.

Physical activity plays an important role in maintaining physical health. As age increases, physical activity levels tend to decline in older adults (51). Chan et al. (52) found that the overall prevalence of physical inactivity among older adults aged 60 and above was 48.8%. Walking may be the primary form of physical activity for older adults, who often prefer to stay at home or walk only in nearby areas, which may lead to insufficient physical activity. The Australian Physical Activity and Sedentary Behavior Guidelines recommend that adults aged 65 and above engage in at least 30 min of moderate-intensity physical activity per week, in addition to daily activities (53). The relationship between physical activity and MCI is complex (54, 55). Most previous studies have reported that physical activity reduces the risk of MCI in older adults (56, 57); this study found that compared to the lowest PAL quartile, participants in the second PAL quartile had a reduced risk of MCI, consistent with the findings of Song (58–61). From a physiological perspective, physical activity not only improves cardiac pumping efficiency, increases cerebral blood flow, and delivers more oxygen and nutrients to brain tissue, promoting brain metabolism and enhancing neuroplasticity, which helps prevent and delay cognitive impairment, but also protects brain function by enhancing the body’s antioxidant defense system, maintaining neuronal structural integrity and brain volume, and improving cognitive abilities in older adults (62–65). Najar et al. (66) conducted a 44-year longitudinal study of 800 Swedish women and found that physical activity reduced the risk of dementia (HR = 0.67). Lam et al. (67) studied the lifestyles of older adults in multiple East Asian countries and their relationship with overall cognition, finding that diverse physical activities were associated with better cognitive status. From the perspective of brain structure and function, physical activity is closely related to changes in gray matter volume, particularly in the hippocampus. Erickson et al. (68) and DiFeo et al. (69) found that physical activity increases hippocampal volume, promotes beneficial changes in the functional activity levels of memory-related cortices (19), and significantly improves spatial memory. Older adults are the group most affected by chronic diseases, and physical activity can reduce the incidence of hypertension, diabetes, and dyslipidemia, as well as the risk of stroke, thereby reducing the risk of MCI.

Physical activity primarily assesses the amount of energy expended during exercise (18). This study found a linear dose–response relationship between physical activity and MCI in older adults. As physical activity increased, the risk of MCI gradually decreased, reaching a beneficial point at 900 MET-min/week, with the lowest risk at approximately 1,600 MET-min/week. Beyond 1,600 MET-min/week, the risk of MCI began to rise, reaching a significant increase at 2,100 MET-min/week, consistent with the findings of Daniel l (70, 71). Low-dose exercise can improve cognitive function in older adults, and short-duration, high-frequency training may have a greater effect on cognitive improvement. Short-duration physical activity is less likely to cause fatigue, and Lam (72) found that moderate physical activities such as Tai Chi and clapping can effectively improve brain function and cognitive abilities in older adults (73). A key parameter in physical activity is exercise intensity. According to the WHO physical activity guidelines, older adults are recommended to engage in 150–300 min of moderate-intensity aerobic activity or 75–150 min of vigorous-intensity aerobic activity per week to enhance cognitive abilities. Zotcheva et al. (33) found that physical activity benefits cognitive function in older adults, but higher intensity and longer duration do not necessarily lead to greater cognitive benefits, and high-intensity physical labor may increase the risk of dementia. Excessive high-intensity physical activity may cause sudden increases and decreases in blood pressure, and long-term repeated stimulation may lead to vascular endothelial damage, potentially accelerating microcirculatory disorders in the brain and promoting MCI. Excessive activity may exceed physiological thresholds, inducing oxidative stress and inflammatory responses, indirectly damaging cerebrovascular function. Excessive high-intensity activity is often accompanied by insufficient rest time, and reduced sleep quality may inhibit the clearance of metabolic waste and synaptic plasticity repair in the brain, accelerating cognitive decline. Excessive high-intensity activity may also lead to musculoskeletal injuries, and chronic pain may activate the hypothalamic–pituitary–adrenal axis, increasing cortisol secretion and directly damaging hippocampal neurons. The psychological stress associated with high-intensity labor may increase glucocorticoid levels, and long-term exposure may damage the prefrontal cortex and hippocampal structures, affecting memory encoding. Another key factor in physical activity is duration, including the duration of each individual activity and the total duration of physical activity. When the duration is 30–60 min, the benefits are greater (58). Effective physical activity duration promotes cerebral blood circulation and redistribution, increases enzyme and pro-inflammatory cytokine activity to enhance antioxidant effects (74), and promotes the production of brain-derived neurotrophic factors, regulates serotonin and kynurenine metabolism, induces myokine interactions in muscle-brain communication, enhances anti-inflammatory responses, and mediates mitochondrial regulation (75). Physical activity reduces Aβ plaque aggregation, promotes neurogenesis and synaptogenesis, and improves brain structure and cognitive neural circuits (76), reduces tau protein aggregation, and increases gray and white matter volume in the hippocampus and temporal cortex, improving learning, memory, and cognitive function (77), and reduces the increased risk of Aβ deposition associated with APOEε4 carriers (78, 79).

Further subgroup analyses found that compared to the second PAL quartile, female participants in the third and fourth PAL quartiles had an increased risk of MCI, consistent with the findings of Baker (80), indicating that physical activity has a greater impact on women. Studies have shown that women’s cerebrovascular regulation mechanisms are more sensitive to pressure changes, and high-intensity activity may lead to fluctuations in cerebral perfusion, exacerbating the risk of neuronal damage‌ (81, 82). Postmenopausal women experience a sharp increase in follicle-stimulating hormone (FSH) levels, which directly activates the C/EBP*β*/AEP pathway, promoting β-amyloid and tau protein deposition. High-intensity activity may exacerbate endocrine fluctuations, further amplifying the neurotoxic effects of FSH‌ (83). High-intensity activity increases energy expenditure, leading to elevated levels of sex hormone-binding globulin, reducing estrogen crossing the blood–brain barrier, and weakening its ability to clear β-amyloid. Women have a stronger ability to release zinc ions from nerve cells, and high-intensity activity may accelerate zinc metabolism imbalance, leading to abnormal zinc ion accumulation and promoting amyloid deposition.

Participants aged 60–74 in the first and fourth PAL quartiles had a significantly increased risk of MCI. Early older adults (60–74 years) are more likely to engage in high-intensity physical activity due to occupational or lifestyle habits, and long-term exposure to mechanical stress or oxidative stress may activate the hypothalamic–pituitary–adrenal axis (HPA axis), promoting β-amyloid deposition and accelerating neuronal damage. Late older adults (75+ years) generally have reduced activity intensity, decreased exposure to stressors, and correspondingly reduced risk. High-intensity activity may exacerbate gut microbiota dysbiosis, reducing the supply of neurotrophic factors (e.g., *ω*-3 fatty acids, B vitamins) through the “gut-brain axis,” affecting neuronal repair. Late older adults have reduced activity intensity, decreased metabolic burden, and reduced risk of related damage. Early older adults’ cardiovascular systems can still withstand a certain level of activity, but long-term high-intensity activity may exceed physiological thresholds, inducing chronic inflammation and vascular endothelial damage, leading to abnormal cerebral blood flow regulation.

Stratified by urban and rural areas and adjusted for multiple confounding factors, it was found that older adults in different residential locations had different impacts on MCI. Rural residents in the fourth PAL quartile had a significantly increased risk of MCI, possibly due to the overall higher incidence of MCI in rural areas compared to urban areas (84, 85). Considering the socio-economic characteristics of rural residents, urban older adults generally have higher education levels and greater ability to receive external information stimulation (86). Long-term high-intensity labor is often accompanied by insufficient rest time, and high-intensity labor may compress cognitive training time, leading to a higher risk of MCI under dual effects‌ (87). Rural older adults may have insufficient intake of key nutrients such as protein and vitamins, and high-intensity activity increases energy expenditure. If nutritional supplementation is not timely, it may lead to energy metabolism disorders in brain cells. High-intensity activity may also interfere with insulin sensitivity, increasing the risk of glucose metabolism abnormalities, which are significantly associated with cognitive decline (88).

This study has some limitations: (1) The study used cross-sectional data from the fifth wave of CHARLS in 2020, during the COVID-19 pandemic, which may introduce information bias; moreover, the cross-sectional design cannot infer causality between physical activity levels and cognitive function changes, which requires further research. (2) The assessment of physical activity levels was based on self-report, which may introduce recall bias. (3) This study only analyzed physical activity levels in older adults, and further in-depth research is needed. Future studies should strengthen longitudinal tracking to explore the optimal timing, cumulative duration, frequency, intensity, and combination of physical and cognitive exercises for reducing the risk of cognitive decline.

## 5 Conclusion

Our findings highlight the benefits of physical activity levels between 900 and 2,100 MET-min/week in reducing the risk of MCI in the Chinese older adult population, with the optimal physical activity level being 1,600 MET-min/week. Physical activity has a greater impact on cognitive function in older women than in older men, in older adults aged 60–74 than in those aged 75 and above, and in rural older adults than in urban older adults. The study provides insights into promoting cognitive health in older adults with MCI.

## Data Availability

Publicly available datasets were analyzed in this study. This data can be found: https://charls.pku.edu.cn/.

## Footnotes

^1^ https://charls.pku.edu.cn/
